# Recent Advances in the Design of Electro-Optic Sensors for Minimally Destructive Microwave Field Probing

**DOI:** 10.3390/s110100806

**Published:** 2011-01-12

**Authors:** Dong-Joon Lee, No-Weon Kang, Jun-Ho Choi, Junyeon Kim, John F. Whitaker

**Affiliations:** 1 Division of Physical Metrology, Korea Research Institute of Standards and Science, Yuseong-gu, Daejeon 305-340, Korea; E-Mail: nwkang@kriss.re.kr (N.-W.K.); 2 The 4th R&D Institute, Agency for Defense Development, Yuseong-gu, Daejeon 305-600, Korea; E-Mails: junhochoi@add.re.kr (J.H.C.); juny@add.re.kr (J.Y.K.); 3 Center for Ultrafast Optical Science, Department of Electrical Engineering and Computer Science, University of Michigan, Ann Arbor, MI 48109-2099, USA; E-Mail: whitaker@umich.edu (J.F.W.)

**Keywords:** microwave measurement, electro-optic measurement, optical sensing, sensors

## Abstract

In this paper we review recent design methodologies for fully dielectric electro-optic sensors that have applications in non-destructive evaluation (NDE) of devices and materials that radiate, guide, or otherwise may be impacted by microwave fields. In many practical NDE situations, fiber-coupled-sensor configurations are preferred due to their advantages over free-space bulk sensors in terms of optical alignment, spatial resolution, and especially, a low degree of field invasiveness. We propose and review five distinct types of fiber-coupled electro-optic sensor probes. The design guidelines for each probe type and their performances in absolute electric-field measurements are compared and summarized.

## 1. Introduction

Over the past two decades, electro-optic (EO) sensing technologies have been continually and viably developed as a practical and unique method for sensing electric fields in a minimally destructive way [1–6]. This is because EO crystals are essentially transparent to both electromagnetic and optical waves. The unique, electrically-transparent aspect of these all-dielectric field probes enables exploration of the near-electric-field distributions of radio frequency (RF) radiators, such as antennas and arrays, or the internal-node diagnosis of high-speed electronic devices and circuits—without disruption to the signals present and without the complicated probe compensation necessary when employing conventional metallic probes. Regarding the electrical transparency of the sensor crystals, both the volume and permittivity (*i.e.*, capacitance) of the material, as well as its supporting embodiment are crucial factors. It is apparent that the design and implementation of EO sensors are crucial to achieving non-destructive microwave sensing applications with suitable sensitivity. One of the most widely used configurations is the mounting of a tiny EO-sensitive crystal tip onto a fiber facet, as this allows the development of all-dielectric embodiments with reasonably small size that minimize distortion of the electric fields to be sensed [7–10].

In this paper, we review a variety of design methods for fiber-coupled EO sensors. Five types of sensors are classified and reviewed by their respective operating principle and probe configuration. Then, the performance of each sensor type is evaluated by characterizing its absolute sensitivity with a standard micro-TEM cell that generates electric-field distributions with accurate, calculable strength for use in probe calibration.

## 2. Design Methods of Fiber-Coupled EO Probes

The earliest schemes for EO sensing used a free-space configuration with a bulk EO crystal itself employed as the sensor [4–6,11–14]. The refractive indices of the EO sensor materials are linearly affected by electric fields that pass through the sensor media. As the properties of the EO-sensor medium along the optical path are modified during exposure to low-frequency electric fields (relative to the optical frequency of the light beam), the light becomes phase-modulated by an additional, field-induced optical phase delay experienced by the part of the light polarized along specific axes of the crystal. The modulated portions of this sensing light beam are eventually demodulated using a photodiode that receives either transmitted or reflected light from the sensor. In practical respects, the reflective scheme is generally preferred, with a sensor tip as the terminal of the optical sensor and the light being modulated at the sensor terminal when it is exposed to an electric field. The reflected beams to be detected are efficiently returned from the sensor using a number of common methods: a mirror [7–9,13–15]; total internal reflection [11–13]; highly reflective surfaces from the device under tests [16]; or through fabrication of a resonator on the sensor plate [10].

**Figure 1.**
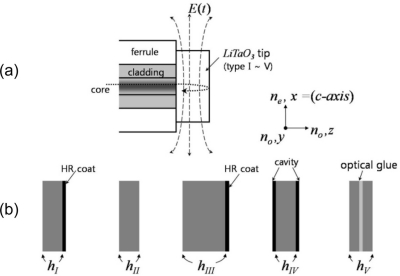
Structure of fiber-coupled electro-optic probes **(a)** direct-mounted reflection style probe and **(b)** five different types of *x*-cut LiTaO_3_ sensor tips.

The most distinguishing feature of EO sensors for use in electromagnetic-field detection is their distinctly low-invasiveness compared to common electrical-field probes. For the bulk probes, interference with the signals to be measured or the operation of the device under test can be substantially decreased, and scanning mobility and spatial resolution can be significantly improved, by mounting the sensor crystals on the output facet of an optical fiber [shown in Figure 1(a)]. Various types of tip-on-fiber EO sensors have been reported [7–10,17,18] and we review and compare five distinct types of EO-sensor designs [shown in Figure 1(b)].

### 2.1. Type I: Conventional Double-Pass Probe

First, we review what is arguably the most common fiber-probe type [7–9]. Here, what is basically a double-pass probe will be called type I. It is based on a retro-reflection process that utilizes a dielectric mirror on the output surface of the probe tip to cause the reflected light to be coupled back along the original fiber path through the tiny fiber core. Such fiber-based EO probes can be categorized into two main sub-types: those that are directly fiber-mounted [7], and those that are attached to a ferrule and GRIN-lens assembly, depending on how the optical beam diverges within the EO crystal [8,9]. The size of the EO crystal is the crucial factor in determining the mounting type, and a clear criterion was presented for this in reference [9]. For thick crystals (up to ∼1 cm), either a quarter- or half-pitch GRIN lens is used to collimate [9] or focus [8] the probe beam onto the sensor plate, respectively. Assembling a probe using a quarter-pitch GRIN lens is generally sufficient to couple the light, but the beam spot becomes maximized at the lens output, while for a half-pitch GRIN the output light is minimized. This is an important design criterion if the spatial resolution of the measured fields is to be optimized.

**Figure 2.**
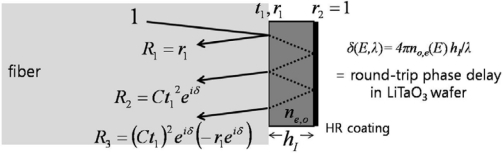
Reflected field components for type I EO probe. The *r*_1,2_ and *t*_1,2_ are, respectively, Fresnel reflection and transmission field coefficients at the front, or incident, and back interfaces. Ideally, *r*_2_ = 1, *t_1_* ≫ *r_1_*, and thus *R_2_* ≫ *R_1_* > *R_3_*. The incident beam is drawn with a non-normal incidence angle for ease in separating the transmitted and reflected beams, (*n*: refractive index, *λ*: light wavelength).

Alternately, crystals up to 1 mm thick can be directly mounted on a thermally-expanded-core fiber-end [7], because the increased numerical aperture of the expanded core allows one to achieve virtually a collimated beam within the crystal volume. As the thickness of a crystal becomes much smaller (to less than 0.1 mm or so), a well-polished fiber ferrule or even a cleaved bare fiber-end is reasonable for capturing the majority of the reflected light. Here, we will concentrate on such a type I probe (*i.e*., ferrule + thin crystal tip with a mirror), as illustrated in Figure 2. In this figure, a 100 μm thick, *x*-cut LiTaO_3_ plate with an HR coat (mirror) on its free surface is mounted directly on the face of a fiber (*n* ∼1.5). The incident optical beam component through the fiber is partially reflected at the first interface (fiber-LiTaO_3_) with the Fresnel coefficient *r_1_*. The transmitted field component (*t_1_*) is then reflected at the second interface (LiTaO_3_-mirror) with another reflection coefficient *r_2_* ∼1.

The majority of this component is coupled back onto the original fiber path. The magnitude of the coupled component is *Ct_1_^2^r_2_*, where *C* is a coupling factor associated with the numerical aperture of the fiber and the thickness of the crystal (where *C* can be maintained at a high value for reasonably thin crystals). This coupled component possesses an additional phase retardation arising from the Pockels effect, given by *δ*(*E,λ*) = *4πn_o,e_*(*E,λ*)*h/λ* and due to the electric (*E*) fields that the EO crystal experiences.

The EO LiTaO_3_ crystal is positive uniaxial (*i.e.*, *n_e_* > *n_o_*) and belongs to the symmetry class *3m*. Hence, it has two birefringence terms, *Δn_o,e_* = (*n_e_* − *n_o_*) + (*n_e_^3^r_33_* − *n_o_^3^r_13_*)*E*/*2* [19]. The first and second terms are the natural and electrically-induced birefringences, respectively. *Δn_o,e_* is the term we can control with *E* fields and polarizations. When the light is linearly polarized along the *e*-axis (or *o*-axis), the phase retardation by the crystal is *δ_e_*(*E,λ*) = *4πn_e_h/λ + 2πn_e_^3^r_33_hE*/*λ* (or *δ_o_*(*E,λ*) = *4πn_o_h/λ* − *2πn_o_^3^r_13_hE*/*λ*), because the light experiences only one refractive index throughout the crystal. In this case, *Δδ_e_*(*E,λ*) = *2πn_e_^3^r_33_hE*/*λ* (or *Δδ_o_*(*E,λ*) = *−2πn_o_^3^r_13_hE*/*λ*) controls the *E*-field-induced phase modulations. Conventional fiber-coupled EO probes sense electric fields in phase modulation forms that are being transformed into amplitude modulations. To realize a conversion to amplitude modulation, additional polarization optics is commonly used to obtain a sine (or cosine)-squared transfer function, created by a pair of crossed polarizers. An optional quarter-wave plate is required if one wishes to shift the operating bias point along the sine-squared curve.

### 2.2. Type II: Interference Probe

In contrast to the conventional single- or double-pass EO-modulation method, it has been demonstrated that for a system employing an interferometric EO probe (here called type II), the use of polarizers and wave plates within the beam path may actually be avoided [17,20]. The type II EO probe utilizes the slope of its interference fringe along with field-induced EO phase retardations, which are inherently created by the EO crystal itself. In the type-I case, the reflections from the two surfaces of the crystal wafer are highly unbalanced (*i.e.*, *r_2_* ≫*r_1_*) due to the mirror, which requires a coating process to produce the last crystal-air interface. In actuality, the two dominant light components—coupled back to the fiber-crystal interface—are *r_1_* + *Ct_1_^2^r_2._exp*(*δ*(*E,λ*)), where *δ*(*E,λ*) can be *δ_e_* or *δ_o_* depending on the polarization of the light beam. Also, *r_1_* and *Ct_1_^2^r_2_* can be comparable because *r_2_* (at the crystal-air interface) is always a bit larger than *r_1_* (at the fiber-crystal boundary), and their difference can be compensated by *Ct_1_^2^* which is accordingly smaller than unity if a crystal with proper thickness and refractive index is used.

**Figure 4.**
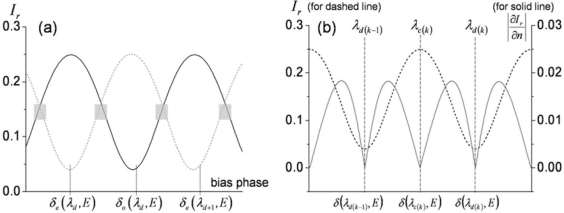
**(a)** Simulated reflectance fringes for type II probe (in Figure 3) *versus* bias phase retardation in terms of wavelength (*λ*: 1550∼1560 nm; *h* = 0.1 mm; solid line: *n_o_* case; dashed line: *n_e_* case; *r_1_*: *fiber-LiTaO_3_*; *r_2_*: *LiTaO_3_-air*; *C =* 1) [17]; **(b)** Total reflectance and reflectance change for 0.01% modulation of *n_e_* (for dashed reflectance in Figure 4(a), with two interference fringes shown).

Since the magnitudes of the two interferometric terms are comparable, clear interference fringes are formed within the range of their sum and difference. The period of the fringes is determined by the phase delay term *exp*(*δ*(*E,λ*)) which is significantly affected by polarization and light wavelength. We use a ∼1.55-μm-wavelength distributed feedback (DFB) laser diode, and the known birefringence parameters of the LiTaO_3_ crystal at 1558 nm are *n_e_* = 2.1224, *n_o_* = 2.1186, *r_33_* = 27.4 (pm/V), and *r_13_* = 6.92 (pm/V) [21]. When a linear beam polarization is set along one of the refractive indices (*n_e_* or *n_o_*) of the LiTaO_3_, the dominant reflected beam components are *r_1_* + *Ct_1_^2^r_2_exp*(*δ_o,e_*(*E,λ*)) (we ignore multi-round trip terms in the crystal, as the first two reflections are dominant). With no exposure to *E* fields from an electronic source, wavelength and polarization are the only quantities that control the reflectance. Figure 4(a) shows the fringe patterns for a 10 nm span near 1.55 μm. Two distinct fringe patterns exit for linear polarizations aligned along the respective *n_o_* or *n_e_* axis of the LiTaO_3_ wafer. Although the amount of natural birefringence (*n_e_* − *n_o_*) is minute, such that either refractive index with a crystal of 100 μm thickness would yield approximately a 5.72-nm mode-spacing at this wavelength band, the position of destructive wavelength (*λ_d_*) could vary substantially, as shown in Figure 4(a). The simulation is done for ideal reflection and coupling conditions (*r_1_*: *fiber-LiTaO_3_*; *r_2_*: *LiTaO_3_-air*; *C* = 1). Such an interference fringe pattern is similar to that of a Mach-Zehnder (MZ) modulator where a DC bias controls the operating point of modulation. Contrary to this, for the type-II interference-based probe, the wavelength serves as the static field bias in the MZ modulators. Setting the spectral biases at the steepest modulation slopes [gray areas in Figure 4(a)], efficient light modulation can be attained by inducing a dynamic phase retardation associated with electric fields. The EO sensitivity is basically proportional to the slope of the fringe pattern. The EO phase modulation (PM) gets transformed to an amplitude modulation (AM) through such a PM-AM conversion curve, and its conversion efficiency slope with respect to the fringe pattern is shown in Figure 4(b).

### 2.3. Type III: Thick Interference-Based Probe

This type of probe can be understood as a hybrid of the type-I and -II probes. As the crystal in a type-II probe becomes thicker, the coupling efficiency *C* degrades, so that the secondary interference term—which contains important EO-phase-modulation information—becomes less associated with the primary interference term that is reflected at the fiber-crystal interface. In type-I probes, this secondary term is dominant over the first term, so the interference effect is often ignored. In type-II probes, those two terms can be reasonably balanced, and thus the PM component may be converted to AM through an interference process without using a polarizer (or analyzer). In either of the type-I or -II cases, it would be greater to increase the EO interaction length by simply employing a thicker crystal. However, the coupling efficiency *C* degrades rapidly due to the beam divergence in a thicker crystal.

The reduced coupling efficiency can be compensated by increasing the reflection at the crystal-air interface (*r_2_*) via an HR-coated mirror. As an example, for a crystal five times thicker than that in Figure 2, the period of the fringes will shrink accordingly due to the increased cavity length, while the coupling efficiencies are nicely maintained by the HR mirror. This will be verified in the experimental section below.

### 2.4. Type IV: Resonance Probe

In the first three types of probes, not only does the conventional type-I case use just a single round-trip phase modulation (based on the double pass scheme of the probes), but so too, essentially, do the other two interference-based probes. To enhance the interaction length of the crystal, there have been numerous investigations that utilize a resonance effect [10,22–28]. Building an optical cavity around the crystal, the probing light can be contained for much longer than in the single-round-trip case, and thus resonantly enhanced cumulative phase components can contribute to an improvement in sensitivity. Moreover, the multi-pass components associated with the cavity can also significantly increase the sharpness of the interference fringes (*i.e.,* the finesse of the cavity). Thus, the PM-AM conversion slope becomes more effective, resulting in sensitivity enhancements without increasing the volume of the crystal.

**Figure 6.**
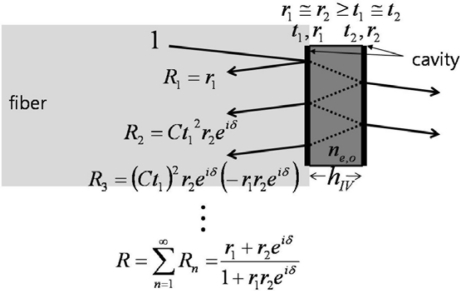
Reflected field components for type IV probe scheme (identical condition to Figure 3 with coatings added to provide better definition for the cavity. Here, *R_1_* > *R_2_* > *R_3_*).

**Figure 7.**
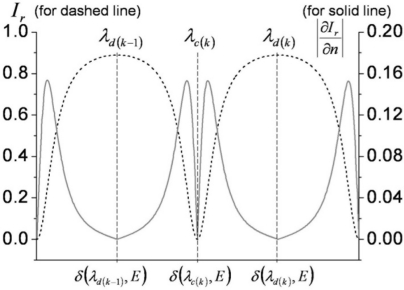
Reflectance (dashed line) and its change (solid line) for type IV probe for a 0.01% modulation of *n_e_*, with two interference fringes shown). (Here, *r_1_^2^* = *r_2_^2^* = 0.5; *C* = 1).

Such resonance-based EO-sensing schemes can be better understood by considering Figure 6. In a cavity that has large reflection coefficients (at least *r* ≥ *t*), higher order reflection terms (*R_3_* or higher) may no longer be ignored, but rather must be included to obtain the overall reflection components. The summation of the reflected field components *R*(*E,λ*) is (*r_1_+ r_2_exp*(*δ*(*E,λ*)))/(1*+ r_1_r_2_exp*(*δ*(*E,λ*))), as expressed in Figure 6. Hence, the reflected intensity is *I_r_*(*E,λ*) = *|R*(*E,λ*)*|^2^*, which heavily depends on the wavelength and polarization of the light. As discussed for the previous probe types, the input polarization is set parallel to the optic-axis to maximize the EO phase retardation and to only capture the light modulation along the *n_e_* axis, while the wavelength bias is set at the point of steepest slope on the spectral fringes. Having fixed these two conditions, the only variable that can change the light intensity is the refractive index of the crystal. Since the EO crystal changes its refractive index with applied *E*-fields, and the amount of index modulation is typically minute for most EO sensing applications, the sensitivity of the EO amplitude modulation is proportional to *∂I_r_*(*E,λ*)*/∂n*(*E*). Figure 7 displays the fringe patterns associated with a cavity (*r_1_^2^* = *r_2_^2^*= 0.5) and its corresponding EO sensitivity.

### 2.5. Type V: Multi-Layer Probe

Building a resonator out of the EO crystal has been a significant advancement for overcoming the inherently minute nature of the Pockels effect on the refractive index of the sensor medium, because of the increase in EO interaction length within the sensor. In the type-IV probe, we used a balanced resonator (Fabry-Perot type) to enhance the EO phase retardation and to allow utilization of the steepened modulation slope. Such resonant retardation could be enhanced even more significantly through the use of an unbalanced structure (*i.e.*, of Gires-Tournois type) [25].

A novel design approach was recently presented for realizing another resonance-based, electro-optic-sensing scheme, without relying on highly reflective surfaces [18]. Utilizing two or more uncoated EO wafers, the reflectance resonance characteristics can be improved, and thus the EO phase retardation and the slope of the function responsible for phase-to-amplitude modulation-conversion (which is also associated with the multi-stratified embodiment) are also enhanced.

The multi-layer scheme is basically an extension of the type-II case, in which a single dielectric EO wafer naturally forms a Fabry-Perot etalon, and thus it has unique transmission/reflection characteristics. The transmittance/reflectance of the etalon is slightly modulated as the refractive index of an EO medium is perturbed by an applied *E*-field. Such EO modulation effects can be enhanced by adding other layers of appropriate thicknesses.

**Figure 8.**
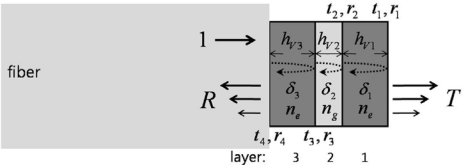
Structure of a multi-layered electro-optic probe (where layers 1 and 3 are thin plates of LiTaO_3_ that yield phase modulations *δ_1_* and *δ_3_*, respectively).

Figure 8 is a schematic of a type-V probe that consists of three stratified layers mounted on a fiber facet. Layers 1 and 3 are *x*-cut LiTaO_3_ wafers, and thus both of their optic-axes are perpendicular to the travel of the optical beam. The incoming optical beam is transmitted through a fiber core (*n_core_* ∼1.5), after which it passes through layer 3 (LiTaO_3_), layer 2 (an optical adhesive with *n_g_* ∼1.5), and layer 1 (identical to layer 3). The overall optical field transmission from such a three-layer system is expressed in Equation 1 based on the transfer function and standard feedback theory [18,29]:

(1) $$T=\frac{t_{4}t_{3}t_{2}t_{1}}{1-(\mathrm{first}+\mathrm{second}+\mathrm{third})\mathrm{order feedback terms}}$$

where the first-order feedback terms are: −(*r*_1_*r*_2_*e*^*iδ*_1_^ + *r*_2_*r*_3_*e*^*iδ*_2_^ + *r*_3_*r*_4_*e*^*iδ*_3_^)
the second-order feedback terms are: −(*r*_1_*r*_3_*e*^*i*(δ_1_+δ_2_)^ + *r*_2_*r*_4_*e*^*i*(δ_2_+δ_3_^) + *r*_1_*r*_2_*r*_3_*r*_4_*e*^*i*(δ_1_+δ_3_)^)
and the third-order feedback terms are: −(*r*_4_*r*_1_*e*^*i*(δ_1_+δ_2_+δ_3_)^)

**Figure 9.**
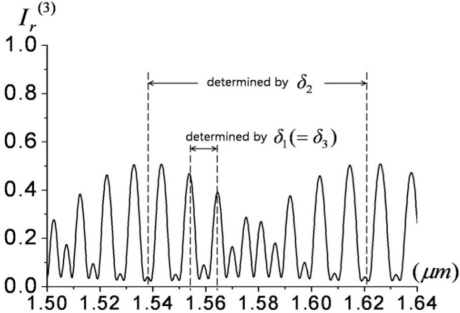
Simulated reflectance fringes for the probe of Figure 8, where *δ_1_*, *δ_2_*, and *δ_3_* are the phase retardances due to layers 1, 2, and 3, respectively [18].

For overall reflectance, we may utilize the complementary relation: *I_r_* = |*R*|*^2^* = *1* − |*T*|^2^ = *1* − *I_t_* for lossless media. The simulated reflectance of this type-V probe for 50-μm-thick EO wafers (layer 1, 3: *n_e_* = 2.1224) and a 10-μm-thick adhesive gap (layer 2: *n_g_* = 1.5) is shown in Figure 9. The polarization of the laser was assumed to be linear along the *x*-direction (extra-ordinary axis) as in previous probe types.

The spectral range chosen was centered at ∼1.55 μm, with a span wide enough to explore complex interferometric fringe relations. The fringes in Figure 9 consist of fine and wide periodic patterns, which are determined by *δ_1_* (or *δ_3_*) and *δ_2_*, respectively. The steeper slope of the reflected-intensity fringes over a broad spectral region is advantageous for efficient EO amplitude modulation. This condition can be achieved by reducing the gap between wafers or by using a gap-medium of lower index. As in previous interference or resonance probes, the sensitivity of the EO amplitude modulation versus a minute *E*-field-induced index, is proportional to *∂I_r_*(*E,λ*)*/∂n*(*E*). Since *I_t_* (*E,λ*) *+ I_r_* (*E,λ*) = *1 − loss*(*λ*), the sensitivity becomes *∂I_r_/∂n* = −*∂I_t_/∂n*, where *n* is an electro-optical variable (here, *n* = *n_1_* = *n_3_* = *n_e_*). The partial derivative of intensity over the variable index can be readily obtained by numerically perturbing refractive index terms. It should be noted that this approach also inherently deals with resonance-based phase-enhancement terms as described in references [23] and [24].

**Figure 10.**
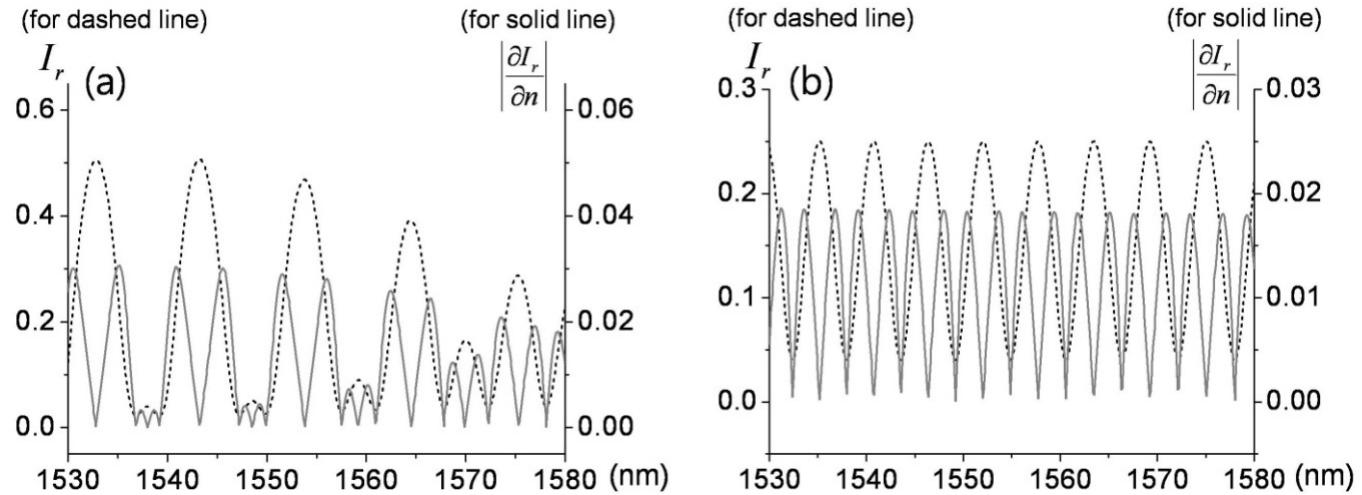
Simulated reflectance fringes and change in reflectance due to refractive index (which corresponds to EO sensing strength) for probe V **(a)** *h_1_* = *h_3_* = 50 *μm*, *h_2_* = 10 *μm*, *n_1_* = *n_3_* = *n_e_*. Part **(b)** is the same as (a) except *h_2_* = 0 *μm* (*i.e.*, probe V becomes probe II) [18].

The reflectance and corresponding EO sensitivity within the wavelength region appropriate to optical-communication sources are computed for 0.01% modulation of *n_e_*, and shown in Figure 10. Figure 10(a) indicates the normalized EO sensitivity compared to the slopes of the reflectance with the simulation parameters as in Figure 9. However, as we remove the gap between the EO wafers (that is *h_1_* = 100 *μm, h_2_* = *h_3_* = 0), the slope of the fringe is reduced, as observed with the correspondingly reduced EO sensitivity in Figure 4(b).

## 3. Performance Evaluation of Fiber-Coupled EO Probes

The set of five probes described above was fabricated in order to conduct an experimental evaluation of their performance. For the conventional, type-I probe, we used a 100 ± 5-μm-thick, *x*-cut LiTaO_3_ plate with a 99% HR-coated mirror. Practically, considering non-ideal coupling conditions (*C* < 1) and minor spectral ripples associated with small reflections at the fiber-LiTaO_3_ interface, the coupled light is typically about two thirds of the input power. Since its spectral slope for effective PM-AM conversion is relatively weak, an additional polarization controller and analyzer were employed just before the photodetector. Here, an analyzer creates the well-known sine-squared PM-AM conversion slope of a single-pass EO modulator, and the second polarization controller adjusts the EO modulation bias. We also made a type-II probe using the same material and procedure, except that the HR mirror was not included.

Using a LiTaO_3_ plate five times thicker than the EO medium of the type-I probe (500 ± 5 μm), a type-III probe was fabricated. A type-IV probe, intended as an improved version of the type II case, was also produced, with a balanced cavity added to the faces of the EO plate. A type-V probe was assembled based on the proposed three-layer configuration in Figure 8. The thickness of each LiTaO_3_ wafer used here was 50 ± 5 μm, and a narrow gap between the two wafers was filled with UV-curing cement. The wafer stack was pressed together in order to minimize the gap between the EO wafers to ∼2 μm.

To utilize the slopes of the spectral reflection fringes in each probe (types II–V), the laser has to cover sufficient wavelength tuning ranges. It would be ideal to use a widely tunable laser source, such as an external-cavity diode laser. However, a commercial optical-communication-grade distributed-feedback (DFB) laser diode can replace the more expensive tunable sources, since a DFB laser exhibits limited tunability via thermo-electric control (TEC). A typical DFB laser diode in the 1.5-μm wavelength band readily covers a few nm of tuning range using such thermal control. The relationship of laser wavelength and temperature control reported previously, where ∼2.5 nm of tuning range was attained by 30 degrees of dynamic temperature control, was again employed in this study [17].

**Figure 11.**
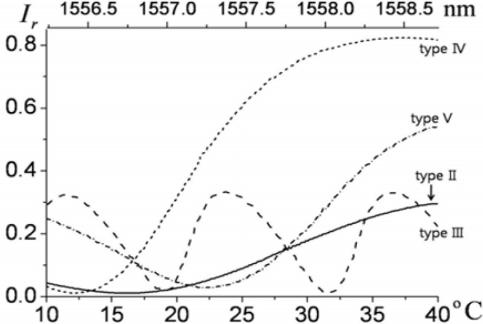
Measured reflectance fringes for probes II through V versus temperature-tuned wavelength within the 1550-nm band.

**Figure 12.**
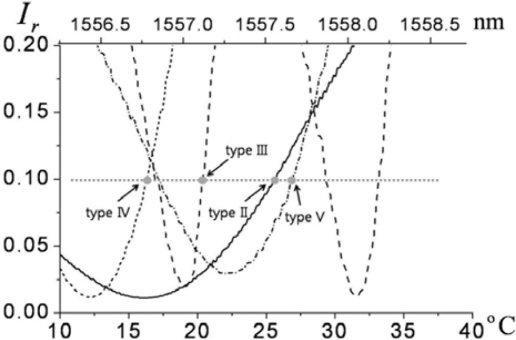
Wavelength bias points (gray dots) for probes II through V in Figure 11 on their respective reflectance fringes. (The biases were set at 10% of the peak reflectances.)

For all of the probes of type II through V, the slopes of the spectral reflection fringes are used in order to perform EO sensing. It is apparent that controlling the wavelength bias (by the laser-diode temperature setting) at the steepest slopes of the fringes yields the strongest EO signal for each probe. However, from a practical perspective, it has been found that it is advantageous to lower the bias to achieve a better signal-to-noise ratio (SNR) [30-35]. This is because the noise level is primarily proportional to the laser power level that a detector receives. Lowering the wavelength bias reduces the light intensity more than the gradient of the fringes. Straightforward modeling and experimental analysis of bias optimization for SNR enhancement can be found in reference [35]. A closer look at the low-bias points for each probe in Figure 11 is presented in Figure 12, where the incident laser power to the probes is 30 mW, and the 10% reflectance level was used for the respective bias point of each probe. The saturation power level of our photodiode is ∼3 mW, and ∼2.8 mW of EO-sensing probe beam is coupled to a photodiode through a circulator. Receiving the same power, the noise level of the photodiode for each probe becomes comparable, whereas the signal strength heavily depends on the slope of the fringes and the interaction length of the crystal. The overall reflectance of each probe (type II–V), measured over 2.5 nm of spectral range, is shown in Figure 11. The polarization of the laser was set along the *n_e_* axis of the LiTaO_3_ wafers, so as to have clean interferometric fringes, as well as maximized EO phase retardation.

**Figure 13.**
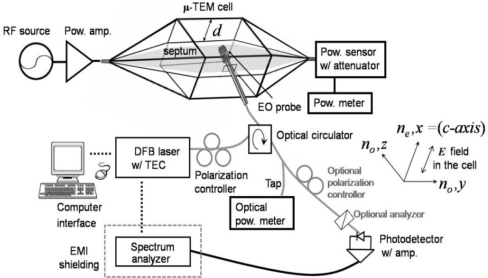
Experimental schematic for an all-fiber-based EO-probe calibration system. (The gray and black lines are optical fibers and electrical connections, respectively).

The sensitivity of each EO probe can be evaluated if one uses a calculable, and thus known electric-field strength from a standard field-generating device. One excellent choice for such field strength calibration is a micro-transverse-electromagnetic (μ-TEM) cell, where the reference field strength can be calculated with low uncertainty from parameters of the cell such as the reflection coefficient, input power, and geometrical dimensions [36–38]. The field strength inside the cell is proportional to the input power and inversely proportion to the septum (center conductor) distance of the cell. The μ-TEM cell has a very small septum height (*d* in Figure 13) compared to a conventional TEM cell, and therefore it can generate a relatively high *E*-field for the same input power level. The electric fields are linearly polarized in a vertical direction in the TEM cell and are uniform in the useable test area, which is located in the center region between the septum and the upper wall of the cell. To sense these fields, the EO probe end was placed at the precise center of the test area through a tiny hole in the side wall. The optic-axis of the sensor is parallel to the *E*-fields (*x*-direction in Figure 13) in order to maximize the sensing fields.

**Figure 14.**
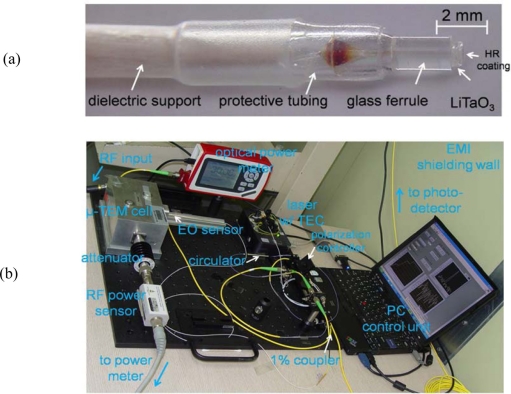
**(a)** Fabricated probe structure (type III); **(b)** actual experimental layout depicted in Figure 13.

The complete probe-evaluation system is illustrated in Figure 13, and a photograph of the actual layout appears in Figure 14.

A fiber-pigtailed diode laser, manual polarization controller, optical circulator, and fiber-connectorized photodiode were spliced together with the probe to form a continuous, low loss, enclosed optical path. The 30 mW of polarization-controlled light flows to the probe-head tip, and the known electric field and the optical beam intersect within the EO medium. The reflected/modulated beam—which contains the valuable information on the amplitude and phase of the electric fields—is routed to the photodetector so that the field components may be demodulated. The SNR performance of such electrical-signal measurements is evaluated using an electrical spectrum analyzer. The μ-TEM cell has broad bandwidth (DC∼1 GHz), and we fed up to +40 dBm of power into the cell at 1 GHz in order to evaluate the high-power-handling capability, linearity, and dynamic range of each probe (type I–V).

The probe-evaluation system requires a second polarization controller and analyzer in the optical beam path before the photodetector for the type-I probe configuration. In conventional type-I sensing, typically the operating bias is set so that the light output is 50% of the input intensity, or at a position of half the maximum value on the sine-squared AM curve. However, similar to the type II-V cases, lowering the bias to a point close to the null output of the modulator is advantageous for SNR, due to the fact that the noise will be suppressed by a greater amount than the signal at this point [30–35]. Maintaining the reflected power from the probe at the same 10% level, the signal level was then maximized by adjusting the two polarization controllers.

**Figure 15.**
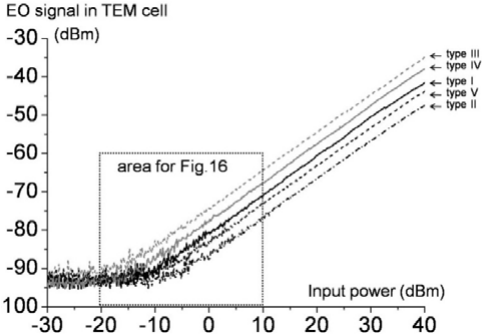
Relative EO performances of the probes, type I through V, in the μ-TEM cell.

**Figure 16.**
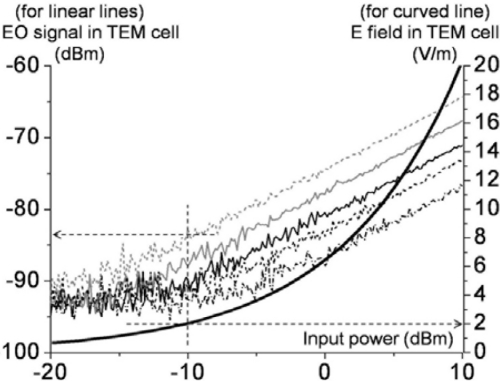
Measured EO signal strength (corresponding to the box in Figure 15) and calculated electric-field strength (thick-solid curve) in the μ-TEM cell versus feeding power. (This relates the measured signals and the calculated fields). The interconnecting lines are guides to the eye.

Compared to previous publications [17,18], an additional preamplifier was used to boost the signal level while significantly lowering the noise level (by ∼15 dB) through shielding of the read-out instrument from electromagnetic interference (EMI). Each signal plot maintains its linearity up to +40 dBm of TEM-cell input power without probe damage or any indication of saturation. Since the EO sensor has an all-dielectric embodiment, it should be able to sense even higher RF power. To investigate the minimum detectable electric fields for the different probes, the detail of Figure 15 at weak RF driving power is considered in Figure 16. By measuring these calculable fields in the standard TEM cell with an EO probe, the EO signal levels can be mapped onto the corresponding absolute field strengths in V/m. The absolute electric field strength within the cell’s test area between the upper wall and the septum (*i.e*., the septum height) is determined by the equation (*PZ_o_*)*^1/2^/d* (in V/m), where *P* is the net power flowing through the cell, *Z_o_* is the complex characteristic impedance of the cell, and *d* is the septum distance [36–38].

Our cell features yield *Z_o_* = 50.319 Ω and *d* = 34.405 mm, and thus by measuring the *P* values versus input power, the calculated absolute electric fields in the test area of the cell can be attained (see the solid curve in Figure 16). For instance, the minimum detectable field of the type III probe (dashed gray line) is determined to be between 1–2 V/m. At −10 dBm of cell power, an EO signal level of ∼6 dB above the upper peak noise level is found. This signal level corresponds to 2 V/m by correspondence to the electric-field plot (solid, curved line). It should be noted that each signal curve is the trace of the peak values on the signal spectra, so the noisy portion along each signal trace is the upper limit of the experiment’s noise floor. The actual average noise floor is ∼−95 dBm ± 5 dB, so the minimum detectable field strength is practically very close to the 1 V/m level. This condition can be achieved averaging the noise level with a longer sweep time on a spectrum analyzer.

The stability of the sensors is an important factor in instances where a long-term measurement is required—for instance, due to the fact that the sensitivities of probe types II–V are all temperature-dependent. Potential temperature drift can be overcome through the use of a TEC-locked loop, which employs feedback so that the wavelength needed for optimal EO sensitivity can be held constant during the entire measurement time. In addition, if one wishes to translate the probes to sense the field at different locations, the light polarization inside the optical fiber should be fixed to insure consistent and optimal operation of the probes. This challenge may be overcome by using polarization-maintaining fibers.

The characteristics of each probe type are summarized in Table 1. The physical operating principle, sensitivity, probe coating, and analyzer requirements have been discussed throughout the text. In regards to fabrication difficulty, the efficient coupling of the reflected beam from the probe crystal back into the optical fiber is the main issue. Probe types I–III are reasonably straightforward to assemble, as these utilize only single round-trip phase-delay components from the crystal. On the contrary, since type-IV probes need to couple multiple round-trip components, and type-V probes utilize sophisticated reflection components resulting from multi-layer coatings, their fabrication is significantly more challenging.

Low invasiveness is one of the distinguishing features of EO sensors. The sensor volume that interacts with the *E*-fields to be measured is a key factor in the invasiveness. For the same sensor volume, the invasiveness of type I, II, and IV probes would be relatively small, since the dielectric optical coating is negligibly thin. The invasiveness increases slightly as the crystals become larger (type III) and/or more complex (type V).

The bandwidth of the sensors is determined by the optical interaction length of the sensor crystal. In case of our type I and II sensors (100 μm, LiTaO_3_), the single round trip optical propagation time is 1.43 ps. Although such delays may be multiplied several times for thicker crystals (e.g., type III) or for longer interaction lengths within cavities (type IV) or periodic structures (type V), the delay should not impact the sensing of microwave fields having the longer temporal periods (e.g., 1 GHz corresponds to a period of 1 ns) examined in these experiments. The bandwidth of the EO crystals themselves is known to extend well into the THz regime [39], so the actual bandwidth of the EO-sensing technique is determined primarily by the probe structure, and secondarily by the capabilities of the high-speed light modulation and detection.

Besides the low invasiveness of the sensors, EO probing is particularly well-suited for high-voltage or high-power microwave applications [40]. In our TEM cell, the max field is 652 V/m at + 40 dBm input power, while the damage level of the all-dielectric EO sensor is known to be on the order of 1 MV/m [40]. We expect these types of probes to have an extremely wide, nearly 120 dB (V/m-MV/m) dynamic range. As the probe handles kV/m-MV/m scales of intense fields, the light would be highly modulated, as much as in a typical electro-optic modulator. Such super intense field information would be readily demodulated using mature optical-telecommunications detection techniques.

## 4. Conclusions

We have reviewed five types of fully dielectric and fiber-coupled electro-optic sensors for non-destructive microwave field detection. The structures and operation principles of conventional double-pass, interference, thick-interference, resonance and multi-layer probes are presented in detail. The performance of each probe is compared by measuring the absolute electric fields with a huge dynamic range. Each type shows pros and cons in various aspects such as sensitivity, cost, invasiveness and sensing speed. The design scheme and performance comparison of each probe can serve as a good guideline for low-invasive and high-power-handling electro-optic sensing applications.

## Figures and Tables

**Figure 3.**
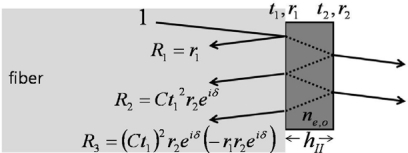
Reflected field components for type II probe scheme (identical condition to Figure 2, except no HR coating. Here, *R_1_* ∼ *R_2_* ≫ *R_3_*).

**Figure 5.**
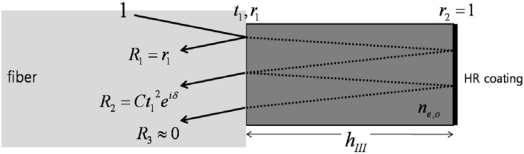
Reflected field components for type III probe (identical condition to Figure 2, except *h_III_* ≫ *h_I_*. Here, *R_1_* ∼ *R_2_* ≫ *R_3_* ≈ *0*).

**Table 1.** Comparison of the five probe types.

**Probe type**	**Probe name**	**Sensitivity**	**Fabrication difficulty**	**Need for coating**	**Need for analyzer**	**Invasiveness**	**Temporal resolution**

I	conventional	good	easy	once	yes	excellent	excellent
II	interference	fair	easy	no	no	excellent	excellent
III	thick-interference	excellent	easy	once	no	fair	good
IV	resonance	excellent	medium	twice	no	excellent	good
V	multi-layer	good	hard	no	no	good	good
